# A novel narnavirus is widespread in *Saccharomyces cerevisiae* and impacts multiple host phenotypes

**DOI:** 10.1093/g3journal/jkac337

**Published:** 2022-12-23

**Authors:** Sriram Vijayraghavan, Stanislav G Kozmin, Wen Xi, John H McCusker

**Affiliations:** Department of Molecular Genetics and Microbiology, Duke University Medical Center, 561 Research Drive 3020, Jones Bldg. Room 239, Durham, NC 27710, USA; Department of Molecular Genetics and Microbiology, Duke University Medical Center, 561 Research Drive 3020, Jones Bldg. Room 239, Durham, NC 27710, USA; Department of Molecular Genetics and Microbiology, Duke University Medical Center, 561 Research Drive 3020, Jones Bldg. Room 239, Durham, NC 27710, USA; Department of Molecular Genetics and Microbiology, Duke University Medical Center, 561 Research Drive 3020, Jones Bldg. Room 239, Durham, NC 27710, USA

**Keywords:** RNA viruses, narnavirus, autophagy, Ras/PKA, 100-genomes strains, *Saccharomyces cerevisiae*

## Abstract

RNA viruses are a widespread, biologically diverse group that includes the narnaviridiae, a family of unencapsidated RNA viruses containing a single ORF that encodes an RNA-dependent RNA polymerase. In the yeast *Saccharomyces cerevisiae*, the 20S and 23S RNA viruses are well-studied members of the narnaviridiae, which are present at low intracellular copy numbers, unless induced by stress or unfavorable growth conditions, and are not known to affect host fitness. In this study, we describe a new *S. cerevisiae* narnavirus that we designate as N1199. We show that N1199 is uniquely present as a double-stranded RNA at a high level relative to other known members of this family in 1 strain background, YJM1199, and is present as a single-stranded RNA at lower levels in 98 of the remaining 100-genomes strains. Furthermore, we see a strong association between the presence of high level N1199 and host phenotype defects, including greatly reduced sporulation efficiency and growth on multiple carbon sources. Finally, we describe associations between N1199 abundance and host phenotype defects, including autophagy.

## Introduction

RNA viruses comprise a large and extremely diversified group. As is the case for many other species, the yeast *Saccharomyces cerevisiae* is a host for RNA viruses. *S. cerevisiae* RNA viruses are vertically transmitted, cytoplasmic genetic elements, which can be divided into 2 families—Totiviridiae (L-A and L-BC, which are encapsidated double-stranded RNA viruses of approximately 4,600 bp) and Narnaviridiae (Wickner *et al*. 2013). Narnaviruses, which have the simplest genome structure of any extant RNA viruses, consist of a single-stranded (ss) monocistronic RNA molecule that encodes an RNA-dependent RNA polymerase (Hillman and Cai 2013). Narnaviruses have been reported in fungal pathogens, such as *Magnaporthe oryzae* (Lin *et al*. 2020), *Alternaria tenuissima* (Nerva *et al*. 2019), *Fusarium* sp. (Osaki *et al*. 2016), and *Aspergillus fumigatus* (Zoll *et al*. 2018), water molds (Hacker *et al*. 2005) as well as in invertebrates, algae, and protozoans (Grybchuk *et al*. 2018; Waldron *et al*. 2018; Goertz *et al*. 2019; Richaud *et al*. 2019). Two well-known members of this family in budding yeast are 20S and 23S (Wesolowski and Wickner 1984; Wickner 1992) that were subsequently characterized by cloning and sequencing their double-stranded (ds) forms W and T, respectively (Matsumoto and Wickner 1991; Esteban *et al*. 1992). The 20S- and 23S-encoded RNA-dependent RNA polymerases (p91 and p104, respectively) form complexes with their single-stranded RNA genomes in a 1:1 stoichiometry in the host cytoplasm (Solorzano *et al*. 2000). 20S and 23S, which have no known phenotypes in host cells, exist in such low copy numbers in vegetatively growing cells as to preclude detection by agarose gel analysis; increased copy numbers are only observed under specific conditions, such as sporulation (Garvik and Haber 1978) and heat shock (Wesolowski and Wickner 1984; Wickner 1992).

Previous surveys of the 100-genomes strains collection revealed extensive natural variation in their nuclear, mitochondrial, and 2-µm genomes (Strope, Kozmin, *et al*. 2015; Strope, Skelly, *et al*. 2015; Vijayraghavan *et al*. 2019). In this study, we identified and characterized a novel narnavirus in *S. cerevisiae*, designated N1199, that we first discovered in 1 of the 100-genomes strains, YJM1199, as a highly abundant double-stranded RNA molecule. We cloned and sequenced N1199 from YJM1199, analyzed its phylogeny relative to other narnaviruses, determined the presence (and RT-PCR genotypes) of N1199 in the other 100-genomes strains, demonstrated cytoplasmic inheritance, and characterized the effects of high copy number N1199 on host phenotypes. Our work demonstrates that a narnavirus, when present in high copy number, can significantly alter host fitness. Our key findings and their implications are described and discussed below.

## Materials and methods

### Strains, plasmids, and PCR primers

The *S. cerevisiae* strains listed in Supplementary Table 1 have been deposited in, and should be requested from, the Fungal Genetics Stock Center <http://www.fgsc.net>. For additional descriptions of the sequenced 100-genomes *S. cerevisiae* strains, or genetic backgrounds, listed in Supplementary Table 1, see Strope, Kozmin, *et al*. (2015), Strope, Skelly, *et al*. (2015), and Vijayraghavan *et al*. (2019). The PCR (and sequencing? ) primers used in this study are listed in Supplementary Table 2. To construct pGAL-*PDE1*, the *PDE1* ORF was amplified from S288C with oligos SV342 and SV343 (Supplementary Table 2), purified, digested with XhoI and NheI (NEB), and ligated into pLND46 digested with SpeI and SalI (NEB). Ligation mixture was transformed into *E.coli*, and transformants were screened for correctly ligated plasmids with restriction digests. The plasmids listed in Supplementary Table 2 have been deposited in, and should be requested from, Addgene (http://www.addgene.org/John_McCusker/).

### Media and phenotypic analysis

YPD and YPE (1% yeast extract, 2% bacto peptone) contained 2% dextrose and 2% ethanol (added after autoclaving), respectively. Similarly, SD and SE (0.67% yeast nitrogen base) contained 2% dextrose and 2% ethanol (added after autoclaving), respectively. YPD, YPE, SD, and SE plates contained 2% agar. Phenotypic analysis was performed on subsets of strains by 10-fold spot dilutions onto 100 mm diameter plates. Media containing other carbon sources at a 2% final concentration were prepared as above. For autophagy induction, cultures were grown in liquid YPD containing 0.1–250 nM rapamycin (StemCell Tech, Cat# 73362) for 2−3 days, and subsequently streaked to YPD plates to allow recovery. Single colonies from the YPD plate were subsequently analyzed for further assays as described below and in the text. For galactose induction experiments, transformants were grown on YP +2% galactose and 100 μg/mL nourseothricin for 2 days and subsequently plated to YPD. Single colonies from YPD plates were analyzed for further experiments as described in the text. The same schematic was followed for analyzing strains transformed with either the test plasmid or an empty vector.

### Virus presence/absence determination by PCR and gel electrophoresis

Total nucleic acids were extracted using a slight modification of the phenol-mediated method described by (Maqueda *et al*. 2010), as follows. Cells of 5-mL overnight YPD cultures were collected by centrifugation and washed once with 50 mM Na_2_EDTA (pH 7.5). Cell pellets were then resuspended in 1 mL of 50 mM Tris-H_2_SO_4_ (pH 9.3)/1% 2-mercaptoethanol solution and incubated for 15 min at room temperature. Cells were subsequently collected by centrifugation and resuspended in 1 mL of 0.1 M NaCl/10 mM Tris–HCl (pH 7.5)/10 mM Na_2_EDTA/0.2% sodium dodecyl sulfate solution. 0.7 mL of phenol (pH 8.0) was added, and samples were incubated on a shaking platform for 1 h at room temperature, after which 0.7 mL of the aqueous phase was recovered by 5-min centrifugation. Nucleic acids were precipitated by the addition of 70 µL of 3 M potassium acetate and 0.7 mL of ice-cold isopropanol, followed by incubation for 5 min at room temperature and centrifugation at 14,000 rpm for 10 min. The precipitated nucleic acids were washed with 70% ethanol, dried using a SpeedVac (Eppendorf), dissolved in 70–100 µL of water, and stored at −80°.

PCR analysis of sequenced RNA viruses was performed as follows. 15 µL aliquots of total nucleic acids samples were incubated for 2 min at 98° and then placed on ice. cDNA synthesis was performed using the Maxima first-strand cDNA synthesis kit (Thermo), in accordance with the manufacturer's protocol, using 5 µL aliquots of nucleic acid samples as a template. PCR reactions were performed with OneTaq DNA polymerase (NEB) in accordance with the manufacturer's protocol. The primers used to detect the presence and, where present, their PCR genotypes, are listed in Supplementary Table 2.

For gel electrophoresis, 5 μL aliquots of total nucleic acid samples (to detect abundant RNA viruses) or RT-PCR-derived nucleic acid samples (to detect PCR products of sequenced RNA viruses) were mixed with 1 × gel-loading buffer and loaded on 1.3% agarose gels prestained with GreenGlo SAFE DNA dye (Denville) and/or ethidium bromide (Bio-Rad) according to the manufacturer's instructions. Electrophoresis was carried out in 1 × TAE buffer at room temperature at a constant voltage of 6 V/cm for 45–60 min. Gels were subsequently imaged under UV light using the Alpha Innotech Red gel documentation system.

### Nuclease treatments

Nucleic acid samples were treated with DNaseI, RNaseI_f_, RNaseH, or ShortCut RNaseIII (all from NEB) at 37°C and samples were analyzed on 1.2% TAE-agarose gel stained with ethidium bromide. For subsequent analyses, treated samples were column purified using standard PCR purification kits (Qiagen), reverse transcribed with Maxima first-strand cDNA synthesis kit (Thermo), and subjected to PCR to test for the presence/absence of various genomic and viral elements, as described above. PCR samples were analyzed on 2% TBE (Tris-borate EDTA) gels at 4–6 V/cm for 75–90 min in prechilled 0.5 × TBE buffer.

### Phylogenetic analysis of the N1199 RNA-dependent RNA polymerase

Sequences were aligned with MUSCLE (v3.8.31) configured for the highest accuracy (MUSCLE with default settings). The phylogenetic tree was reconstructed using the maximum likelihood method implemented in the PhyML program (v3.1/3.0 aLRT). The WAG substitution model was selected assuming an estimated proportion of invariant sites (of 0.002) and 4 gamma-distributed rate categories to account for rate heterogeneity across sites. The gamma shape parameter was estimated directly from the data (gamma = 1.211). Reliability for internal branch was assessed using the aLRT test (SH-Like). Graphical representation and edition of the phylogenetic tree were performed with TreeDyn (v198.3) (Guindon and Gascuel 2003; Edgar 2004; Anisimova and Gascuel 2006; Chevenet *et al*. 2006; Dereeper *et al*. 2008, 2010).

### Cloning and sequence determination of N1199 from YJM1199

Full-length sequence of the N1199 dsRNA from YJM1199 was determined by various modifications to the previously described FLAC (full-length amplification of cDNA) method (Maan *et al*. 2007; Nomikou *et al*. 2009; Potgieter *et al*. 2009; Vepstaite-Monstavice *et al*. 2018; Sanchez *et al*. 2019). The 2.6 kb dsRNA from YJM1199 was gel-extracted, purified, and ligated to the PC3-T7 loop primer (5′ P-GGATCCCGGGAATTCGGTAATACGACTCACTAT ATTTTTATAGTGAGTCGTATTA-3′). Approximately 1 μg of the dsRNA was ligated to 250 ng PC3-T7-loop primer in the presence of 20% PEG-6000 (VWR), 0.01% BSA, 1% DMSO, 20U RiboLock RNase inhibitor (Thermo), 5U T4 RNA Ligase (Thermo), 0.5 μL T4 DNA Ligase (NEB) and 2 μL each of T4 RNA and DNA ligase buffers in a total volume of 40 µL. The ligation reaction was carried out at 37° for 40 min, followed by incubation at 4°C.

The oligo-ligated dsRNA was subsequently purified using the NucleoSpin Gel and PCR Clean-up kit (Machery-Nagel) and denatured at 99° for 3 min in the presence of 1 M betaine (Sigma) and 3% DMSO. cDNA synthesis was carried out using ∼ 300 ng denatured oligo-ligated RNA with M-MuLV reverse transcriptase (RNaseH minus; NEB) for 5 min at 25° followed by 60 min at 42° and 20 min at 65°. RNA removal was performed by adding 5U RNaseH (NEB) directly to the cDNA reaction, followed by incubation at 37° for 25 min and 95° for 5 min. The primary cDNA strands were annealed without further purification by incubating the above mix at 95° for 4 min, and by a gradual lowering of temperature to 80° at the rate of 1°/min, 50° at the rate of 3°/min and finally to 4° at 0.1°/min.

PCR amplification of the cDNA was carried out using Phusion DNA polymerase with PC2 primer (5′-CCGAATTCCCGGGATCC-3′) per the manufacturer's protocol, with a single additional 2 min incubation step at 72° immediately following initial denaturation. PCR products were analyzed on a 1%TAE-agarose gel. The 2.6 kb PCR amplicon from the previous step was phosphorylated with polynucleotide kinase and blunt-ligated into pAG25 (Goldstein and McCusker 1999) linearized with EcoRV and dephosphorylated with CIP, to yield plasmids pSV35 and pSV36. Clones were analyzed by restriction digests and sequencing. All enzymes were obtained from NEB unless noted otherwise. The full sequence of the cloned cDNA was determined by primer walking and aligning overlapping sequences *in silico* using the SnapGene software (from GSL Biotech; available at snapgene.com). DNA and protein sequence homologies were determined using NCBI Blast (https://blast.ncbi.nlm.nih.gov/Blast.cgi).

### Cell fractionation experiments

Cell fractionation was performed according to Keogh et al. (2006). Cells from 50 mL overnight YPD cultures of YJM1199 were collected by centrifugation, washed with ddH_2_O, and subsequently with PSB (20 mM Tris-Cl pH 7.4, 2 mM EDTA, 100 mM NaCl, 10 mM β-mercaptoethanol) and SB (1 M sorbitol, 20 mM Tris-Cl pH 7.4). Cells were treated with 10 mg/mL Zymolyase 20T in 1 mL SB at 30°C with rotation until spheroplasts were observed. Spheroplasts were harvested at 2000*×g* for 5 min at 4°C, washed twice with SB, and resuspended in 500μL EBX (20 mM Tris-Cl, pH 7.4, 100 mM NaCl, 0.25% Triton X-100, 15 mM β-mercaptoethanol and 20 mM phenylmethylsulfonyl fluoride). To lyse the plasma membrane, the suspension was treated with 0.5% Triton X-100 and incubated on ice with gentle mixing for 10 min, after which a small aliquot of the lysate was removed for gel analysis and the remainder layered over 1 mL NIB (20 mM Tris-Cl pH 7.4, 100 mM NaCl, 1.2 M sucrose, 15 mM β-mercaptoethanol, and 20 mM phenylmethylsulfonyl fluoride) and centrifuged at 12,000*×g* for 15 min at 4°C. The upper layer containing the cytoplasmic fraction was stored for gel analysis, while the pellet containing the nuclear fraction was resuspended in 500μL EBX and treated with 1% Triton X-100 to lyse the nuclear membrane. After 10 min of incubation on ice, the sample was spun down as previously to yield a supernatant containing the nucleoplasm fraction and a chromatin-containing pellet. The pellet was washed with EBX 3 times and resuspended in 50 μL Tris pH 8.0. Each fraction was phenol:chloroform extracted to remove proteins and lipids followed by nucleic acid precipitation with 3 M potassium acetate and isopropanol. All fractions were analyzed on 1.3% TAE-agarose gels as described earlier.

### Determination of N1199^hi^ frequency and loss rates

Seven independent colonies of YJM1199 N1199^hi^ grown on YPD plates were scraped off and resuspended in water, generating 7 independent cell suspensions. Appropriately diluted aliquots of each suspension were plated on selective maltose-containing plates (to determine the number of Mal^+^ cells in a colony) and control dextrose-containing plates (to determine a total number of viable cells in a colony) and incubated for 2–3 days at 30°C. N1199 loss frequencies (*f*) were calculated as a ratio of Mal^+^ cells in a colony to the number of total viable cells in a colony. Using median *f* value and median total number of viable cells in a colony (*N*_t_), N1199 loss rate per cell per generation (*m*) was calculated by Drake's equation: m = *f*/ln(*N*_t_•*µ*) (Drake 1991). 95% nonparametric confidence limits for median *f* value was calculated per (Altman 1991) and transformed to rate confidence limits using Drake's equation.

### Curing of the mitochondrial genome from YJM1199

YJM1199 cells were passaged twice in YPD containing ethidium bromide (first in 25 mg/L for 6 h, then in 10 mg/L for 12 h), plated to YPD, and then tested for petite phenotype on YPEG (2% ethanol, 2% glycerol). Petites were confirmed as *ρ*^0^ by DAPI staining (Williamson and Fennell 1979).

### GFP-Atg8 fluorescence microscopy

The plasmid pSV58 was generated by subcloning a 2.6 kb NsiI-EcoRI fragment from GFP-ATG8(416)/GFP-AUT7(416) (Addgene plasmid #49425) into a pAG36 backbone linearized with NsiI-EcoRI digestion create an episomal vector with a NAT marker expressing GFP-Atg8 under the control of the endogenous Atg8 promoter. Transformants were selected on YPD containing 100 μg/mL nourseothricin. The assay was performed as described in Torggler et al. (2017) with modifications. Cultures of YSV914 and YSV918 were grown overnight in YPD were subcultured at a density of roughly 1.5•10^7^ cells/mL to SD, SD-N (lacking a nitrogen source), and SD + 100 nM rapamycin media and incubated with shaking at 30°C for 48 h. All cultures contained 100 μg/mL nourseothricin throughout the experiment for plasmid maintenance. 20 μg/mL phenylmethylsulfonyl fluoride (PMSF, Sigma-Millipore) was added to cultures to prevent vacuolar degradation and increase the intensity of the GFP signal. Since ammonium sulfate can interfere with aminoglycoside antibiotics, L-glutamate (monosodium salt) was instead used as a nitrogen source in SD cultures (Cheng *et al*. 2000). For fluorescence microscopy, an aliquot of the cultures was spun down at 3000 × *g* for 5 min, washed in dH_2_O once, and resuspended in water. 5 μL of the cell suspension was applied to poly-L-lysine coated glass slides, sealed with a coverslip, and imaged with a Zeiss AxioObserver inverted fluorescence microscope with a mounted CCD camera using a 63 × oil-immersion objective using the appropriate DIC and fluorescence filters. Images were pseudo-colored to highlight the GFP signal.

### Statistical methods

Graphical data analysis was performed using GraphPad Prism version 8 for macOS, GraphPad software (La Jolla, CA, www.graphpad.com). Data were plotted as the mean and standard deviation of 3 independent replicates. Statistical significance was analyzed by 2-way ANOVA correcting for multiple comparisons using statistical hypothesis testing (Sidak test). Multiplicity-adjusted *P*-values were reported for each comparison.

## Results and discussion

### An abundant, predominantly double-stranded RNA in YJM1199

**Fig. 1.**
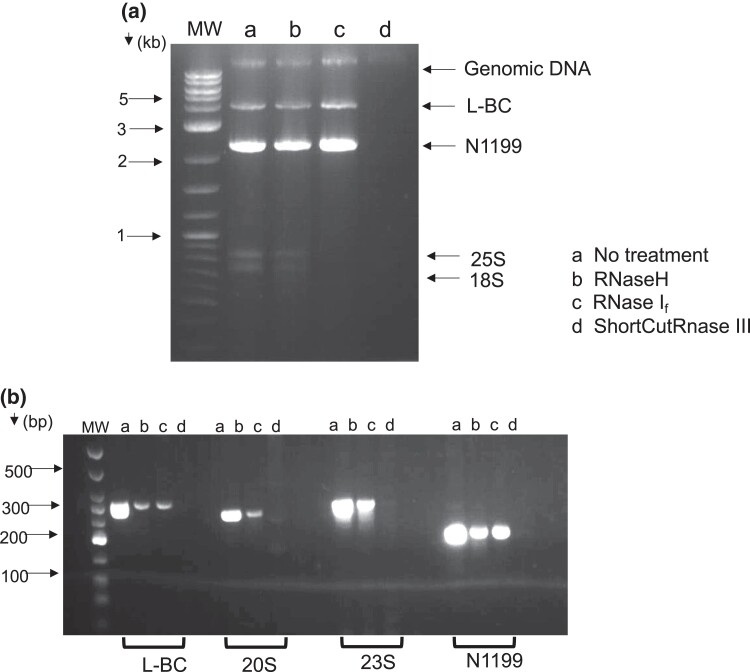
Nucleic acid analysis of N1199. a) Nucleic acid samples treated with the above enzymes per manufacturer's recommendations and run on a 1.3% TAE-agarose gel. rRNA species are indicated. b) PCR products for the indicated species were analyzed on a 3% TBE-agarose gel. Primers used for PCRs: L-BC–L-BC F2 + R2, 20S–RW307 + RW308, 23S–23S F2 + R2, N1199− SV231 + 232 (Supplementary Table 2).

By RT-PCR with multiple primer pairs, the homothallic diploid strain YJM1199 was found to be L-A^0^, L-BC^+^, 20S^+^, and 23S ^+^ . By agarose gel electrophoresis, 2 prominent RNA species were observed in YJM1199—a 4.6 kb band, consistent with L-BC, and a highly abundant 2.6 kb band of unknown identity (Fig. 1). Among the 100-genomes strains, a highly abundant 2.6 kb gel band was observed only in YJM1199 (referred to as N1199^hi^ throughout the text).

**Fig. 2.**
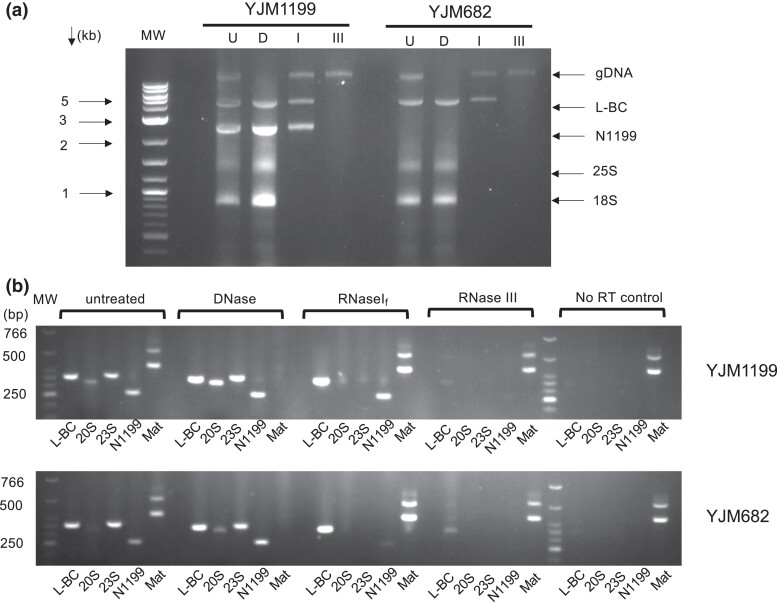
Nuclease treatment and PCR analysis of YJM1199 and YJM682. a) Nucleic acid samples treated with DNaseI (D), RNaseI_f_ (I), or ShortCut RNaseIII (III). U-untreated sample. 25S and 18S rRNA species are single-stranded RNA (ssRNA) species commonly observed in gel analysis of nucleic acids from *S. cerevisiae* strains. gDNA-genomic DNA. b) Samples from (a) were reverse transcribed and analyzed for the presence/absence of the following nucleic acid species: L-BC, 20S narnavirus, 23S, N1199, and MAT locus (as a metric for the presence of intact genomic DNA). No RT control indicates PCRs carried out on control samples reverse transcribed without the addition of the reverse transcriptase enzyme. Primer pairs that produced comparable amplification of the given species for the 2 parental strains were chosen for PCR analyses and were as follows: L-BC–L-BC F2 + R2, 20S–RW307 + RW308, 23S–23S F2 + R2, N1199– SV231 + 232, Mat locus–SV155 + SV156 + SV157 (Supplementary Table 2). MW-DNA molecular weight marker. PCR samples were analyzed on 2% TBE gels. RNaseIII = ShortCut RNaseIII.

Purified nucleic acid from YJM1199 was subjected to various nuclease treatments (Fig. 1). Treatment with DNaseI did not change the abundance of the 4.6 kb or 2.6 kb bands, consistent with both species being RNA molecules (Fig. 2). To rule out RNA–DNA hybrids, we also tested with RNaseH, which specifically hydrolyzes RNA strands in RNA–DNA hybrids and observed no change in either RNA species. In contrast, RNaseIII treatment eliminated all RNA species. To test whether the 4.6 and 2.6 kb RNA species were single- or double-stranded, we treated samples with RNaseI_f_, which is specific for ssRNA, and found that both the putative 4.6 kb L-BC band and the 2.6 kb band were unaffected. In contrast, known ssRNA species including the ribosomal RNAs, as well as the single-stranded narnaviruses 20S and 23S, were depleted by RNaseI_f_ treatment, as confirmed by agarose gel and RT-PCR analyses, respectively (Fig. 1a, b). We concluded that the highly abundant 2.6 kb species in YPD-grown YJM1199 is dsRNA.

### The abundant dsRNA in YJM1199 is a novel narnavirus, designated N1199, that is widespread within the 100-genomes strains as a low abundance ssRNA that varies in sequence

We utilized a modified FLAC cloning methodology (Potgieter *et al*. 2002, 2009; Maan *et al*. 2004, 2007) (Supplementary Fig. 1) to isolate, reverse transcribe and clone the abundant dsRNA in YJM1199; we then sequenced the clone end-to-end with flanking and nested primers. In doing so, we identified a single 2589 bp ORF encoding a putative RNA-dependent RNA polymerase (RdRP). A protein BLAST search with the N1199 putative RdRP-encoding ORF revealed 38% similarity between N1199 and 20S (95% query coverage) and 31% similarity between N1199 and 23S (54% query coverage). Phylogenetic analysis of the N1199-encoded putative RdRP showed its proximity to that of the 20S narnavirus, the recently identified I-329 narnavirus (Mardanov *et al*. 2020), as well as to that of a *Rhizopus* sp. narnavirus (Supplementary Fig. 2).

Based on the N1199 sequence information, we designed multiple primer pairs to perform an RT-PCR analysis of N1199 in the 100-genomes strains (Supplementary Tables 2 and 3). As an initial test, a side-by-side comparison of YJM1199 with YJM682 revealed that N1199 could be successfully amplified from both strains, despite the lack of a prominent 2.6 kb gel band in YJM682 (Fig. 2). Nuclease treatments of RNA from the 2 strains showed equivalent effects on N1199, except for RNaseI_f_ treatment where a marked depletion was noted in YJM682, suggesting that N1199 can exist at a low level as a predominantly single-stranded species in other strains, like the 20S and 23S narnaviruses.

As stated above, in contrast to the parental YJM1199 that has a high level of N1199 (N1199^hi^), none of the 99 remaining 100-genome strains exhibited a high abundance 2.6 kb gel band. Therefore, we used RT-PCR with 5 primer pairs (see Supplementary Table 2 for primer sequences and N1199 sequence coordinates) to test for the presence of N1199 in these strains. While there was strain-specific variation in the level of amplification with some primer pairs, we detected N1199 amplicons with at least 1 of the 5 primer pairs in all but 1 strain (Supplementary Table 3), consistent with 98 of the 100-genomes strains carrying N1199 at a low level (N1199^lo^); that is, N1199^lo^ is not visible via total nucleic acid gel but is detectable by RT-PCR while N1199^0^ is detectable by neither total nucleic acid gel nor by RT-PCR. BLAST analysis of the original genome sequences of the 100-genome strains showed no DNA sequences with similarity to N1199; thus, widespread genomic N1199 cDNA(s) were excluded as a source of N1199 PCR products. Based on the presence/absence of amplicons from the 5 primer pairs, which is hypothesized to be due to N1199 sequence variation, there were 8 RT-PCR N1199 genotypes (Supplementary Table 3).

### N1199^lo^ is cytoplasmically inherited and N1199^hi^ is not affected by treatments that cause the loss of amyloid prions or mitochondrial DNA but is lost with SKI1 overexpression

We were unable to test the cytoplasmic inheritance of N1199^hi^ because all YJM1199 N1199^hi^ segregants were N1199^0^ (see below). Instead, we tested the inheritance of N1199^lo^ by crossing YSV698 (isogenic with YJM682 (L-A^0^ L-BC^+^ 20S^+^ 23S^+^ N1199^lo^)) with YSV706 (isogenic with YJM1463 (L-A^+^ L-BC^+^ 20S^0^ 23S^0^ N1199^0^)), sporulating the resulting diploid, and analyzing spore clones from independent tetrads (Supplementary Fig. 3). Using RT-PCR, in addition to 4:0 inheritance of L-A, 20S and 23S, we observed 4:0 inheritance for N1199^lo^, consistent with the cytoplasmic transmission that is typical of yeast viruses. We confirmed the cytoplasmic localization with cell fractionation of YJM1199 N1199^hi^ samples, whereby N1199 RNA was exclusively present in the cytoplasmic fraction but not in the nuclear fraction (Supplementary Fig. 4). To assess the possibility that N1199 is a mitochondrially localized narnavirus that requires mitochondrially encoded gene products for its replication (Hillman and Cai 2013), we made ρ^0^ derivatives of YJM1199 and found that these still contained high levels of N1199 (Supplementary Fig. 5a).

Like RNA viruses, prions are cytoplasmically inherited (Wickner 1994, 1996; Nakayashiki *et al*. 2005; Wickner *et al*. 2013). Thus, we tested the hypothesis that N1199^hi^ maintenance or abundance is affected by the presence of amyloid prions. Treatment of yeast strains with the chaotropic salt guanidine hydrocholoride (Gdn-HCl) results in the rapid elimination of [PSI + ] (Tuite *et al*. 1981; Eaglestone *et al*. 2000) and other amyloid prions (Wickner *et al*. 2015). However, N1199 levels remained high in YJM1199 after treatment with 5 mM Gdn-HCl (Supplementary Fig. 5b). Given the effects of N1199^hi^ on carbon source utilization, in particular glycerol, that are described below, we also tested for the presence of the [GAR + ] prion that affects carbon source utilization (Brown and Lindquist 2009; Jarosz *et al*. 2014). However, neither N1199^hi^ nor N1199^0^ YJM1199 background strains exhibited the ability to grow on glycerol in the presence of glucosamine, a [GAR + ] phenotype (Supplementary Fig. 5c).

*SKI/XRN1* is an exosome-associated RNA exonuclease, the overexpression of which previously has been shown to cure *S. cerevisiae* strains of L-A; curing of 20S was only seen when its 5′-stem structure was weakened (Esteban *et al*. 2008). Upon galactose-mediated overexpression of *SKI1,* while there was no effect on L-BC by gel, we consistently observed loss of N1199^hi^ in multiple isolates, as assessed by gel analysis, often accompanied by the co-curing of 20S and 23S, as assessed by RT-PCR (Supplementary Fig. 6a). In contrast, when strains carrying an empty vector were similarly induced with galactose, we did not observe a difference in N1199 gel abundance (Supplementary Fig. 7).

### N1199^hi^ is associated with sporulation and multiple carbon source utilization defects

**Fig. 3.**
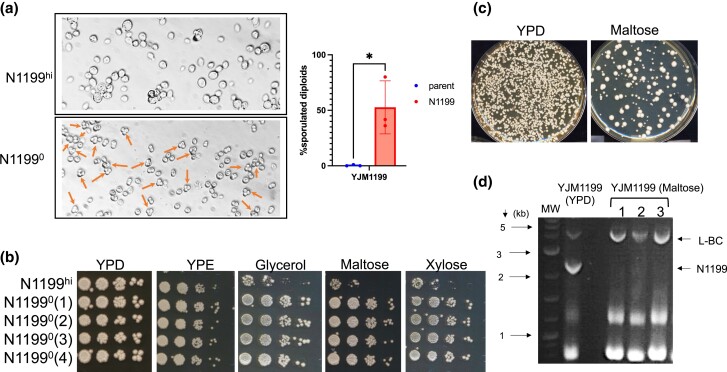
Phenotypic comparisons of isogenic YJM1199 background N1199^+^ vs N1199^0^ strains. a) Comparison of sporulation efficiencies of YJM1199 derivatives containing (top, N1199^hi^) or lacking (bottom, N1199^0^) N1199; arrows indicate asci. MW-DNA molecular weight marker. Quantification of sporulation efficiency of N1199^hi^ vs N1199^0^ isolates of YJM1199 shown with an asterisk denoting a significant difference in means. b) Spot dilutions on media containing different carbon sources. Top lane is the parental YJM1199 containing high-abundance N1199 (N1199^hi^). The next four lanes are spore clones from a single tetrad of sporulated YJM1199, each of which has lost N1199 (N1199^0^, YSV 765–768, Supplementary Table 1). c) Plate assay comparing the growth of YJM1199 on media containing dextrose (left) or maltose (right) as the sole carbon source. Approximately 1,000 cells were plated on each plate and growth was recorded after 2–3 days. d) Representative gel showing RNA samples from colonies isolated from growth on maltose. Image has been cropped to only display a subset of samples (1–3, YSV790–792, Supplementary Table 1).

We previously noted poor sporulation efficiency for YJM1199 under multiple conditions (Strope, Skelly, *et al*. 2015). Interestingly, we observed a complete loss of N1199 (N1199^0^) in all YJM1199 spore clones, both by agarose gel analysis and RT-PCR (Supplementary Fig. 6b), a counter-intuitive result for a yeast RNA virus. Furthermore, while there was no loss of L-BC, the loss of N1199 was accompanied by a concomitant loss of 20S and 23S narnaviruses from the spore clones (Supplementary Fig. 6). The poor sporulation of the parental N1199^hi^ YJM1199 was alleviated in the *HO* N1199^0^ derivatives (Fig. 3a).

We previously observed growth defects for parental YJM1199 N1199^hi^ on different carbon sources (Strope, Skelly, *et al*. 2015). Thus, we compared the carbon source utilization phenotypes of isogenic N1199^hi^ and N1199^0^ YJM1199 derivatives by plating 10-fold dilutions of cells on media containing different carbon sources. The parental YJM1199 N1199^hi^ exhibited poor growth on media containing disaccharides (maltose, melibiose, cellobiose), trisaccharides (raffinose, melezitose), pentose sugars (xylose, arabinose), and glycerol. In contrast, isogenic N1199^0^ derivatives showed markedly improved growth (Fig. 3b, Supplementary 8). Compared to YPD, cultures plated on YP with nonpreferred carbon sources had drastically reduced CFUs (Fig. 3b, c, Supplementary Fig. 9). When tested by gel analysis, 3 Mal^+^ derivatives of YJM1199 (YSV790–792; Supplementary Table 1) were no longer N1199^hi^ (Fig. 3d). When 4 Mal^+^ derivatives with no N1199 band visible on a gel were tested by PCR, all were N1199^lo^, with 2 being 20S^+^ 23S^0^ and 2 being 20S^0^ 23S^+^ (Supplementary Fig. 8b). Using fluctuation analysis (Drake 1991), we estimated the rate of loss of N1199^hi^ per generation to be 7.2 × 10^−4^ (Supplementary Table 4).

### N1199^hi^ is associated with mis-regulated Ras/PKA signaling and autophagy defects

Previous studies in *S. cerevisiae* have shown that elevated Ras/PKA signaling is linked to increased catabolite repression of maltose genes and consequently severely reduced growth on maltose (Wanke *et al*. 1997). Similarly, elevated PKA signaling is conducive for vegetative growth whereas low levels are necessary for entry into meiosis (Jungbluth *et al*. 2012).

**Fig. 4.**
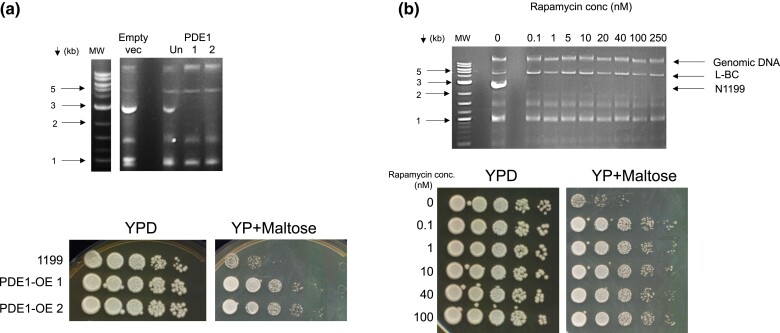
Effects of rapamycin and Pde1 overexpression on N1199. a) Galactose-regulated *PDE1* was overexpressed in YJM1199 under Gal control and 2 independent colonies were analyzed (1,2). Empty vec. indicates vector control with galactose induction. Un-uninduced *PDE1*. N1199-cured isolates were tested phenotypically.1-YSV860, 2-YSV861 (Supplementary Table 1). b) YJM1199 cultures were incubated with 0–100 nM rapamycin for 4 days, allowed to recover on YPD, and checked for the presence/absence of N1199. Strains cured of N1199 at 5 different concentrations were tested phenotypically (1,199 Rapa 0.1–100nM-treated, YSV869-873, respectively).

To test whether the growth phenotypes seen in YJM1199 are connected to the Ras/PKA pathway, we overexpressed *PDE1*, which encodes a low-affinity cAMP phosphodiesterase, in the YJM1199 background (pSV55, Supplementary Table 2). Overexpression of *PDE1* has been shown to decrease the intracellular levels of cAMP in yeast (Wera *et al*. 1997; Ma *et al*. 1999). YJM1199 isolates overexpressing *PDE1* were indeed able to grow normally on media containing maltose (Fig. 4a). Upon testing, we found that *PDE1* overexpression cured N1199 and resulted in similar phenotypes as observed with other N1199-curing experiments (Fig. 4, Supplementary Fig. 10a).

We hypothesized that the hypothetical Ras/PKA-N1199 level association may affect autophagy. Various studies have demonstrated that increased Ras signaling essentially blocks autophagy in yeast (Abeliovich and Klionsky 2001; Natarajan *et al*. 2001; Huang and Klionsky 2002; Budovskaya *et al*. 2004; Stephan *et al*. 2009). Thus, we asked if autophagy induction affects the copy number and/or maintenance of N1199^hi^. To test this hypothesis, we used subcytostatic concentrations of the TOR inhibitor Rapamycin, which has been shown to induce autophagy in yeast strains even under nutrient-rich conditions (Abeliovich and Klionsky 2001; Lu *et al*. 2016; Noda 2017). To test the effects of rapamycin on N1199, cultures of YJM1199 (N1199^hi^ 20S^+^ 23S^+^) were grown to stationary phase in minimal media containing 0–250 nM rapamycin, followed by outgrowth on YPD. The resulting isolates were subsequently analyzed to monitor the effects on N1199 RNA abundance. We found that even subnanomolar concentrations of rapamycin-induced N1199 loss, with all the N1199-cured cultures having phenotypes like those produced in other experiments (Fig. 4b, Supplementary Fig. 10b). Like our previous experiments, in addition to the loss of N1199, we also were unable to detect 20S and 23S by RT-PCR in the rapamycin-treated strains, consistent with co-curing of all 3 narnaviruses (Supplementary Fig. 10b).

**Fig. 5.**
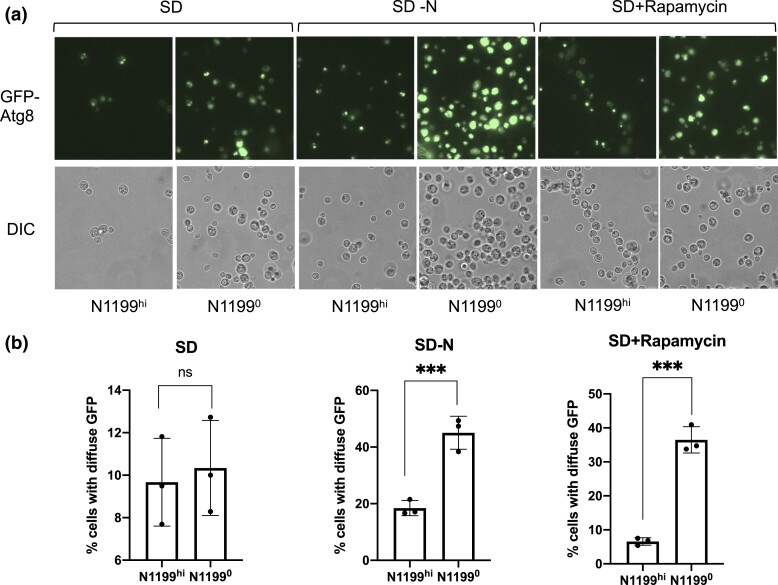
Measurement of autophagy by GFP-Atg8 localization. Fluorescence microscopy of cells from YJM1199 (N1199^hi^) and an isogenic N1199-cured strain (N11990, YSV860, Supplementary Table 1) under various treatments. Graph is presented as mean values with standard deviation, and * indicates statistical significance based on unpaired 2-tailed *t*-tests. *P*-values <0.05 were deemed statistically significant (marked by ** or ***) for shown comparisons. SD-N – SD media without a nitrogen source.

We extended our analysis of the autophagy defect in N1199^hi^ YJM1199 by monitoring the localization and vacuolar processing of Atg8, a ubiquitin-like protein with a regulatory role in autophagosome formation and the cytoplasm-to-vacuole targeting (CVT) pathway (Yorimitsu and Klionsky 2005; Geng and Klionsky 2008; Xie *et al*. 2008). Induction of autophagy results in the delivery of cytoplasmic Atg8 to the vacuole, where it is degraded by vacuolar proteases (Kirisako *et al*. 1999; Huang *et al*. 2000). Using a N-terminal GFP tagged Atg8, bulk autophagy can be quickly and easily monitored via fluorescence microscopy by measuring the change in the intensity of GFP signal within cells (Suzuki *et al*. 2001). In nutrient-rich conditions, both N1199^hi^ and N1199^0^ strains had low levels of autophagy (SD, *P*-value 0.7220). In contrast, under conditions of nitrogen starvation, N1199^hi^ cells displayed poor enrichment of the GFP signal compared to N1199^0^ isolates (SD-N, *P*-value 0.002), with substantial vacuolar fragmentation (Fig. 5). A similar effect was seen when autophagy induction was monitored after rapamycin treatment, whereby there was a substantial loss of GFP signal in YJM1199 N1199^hi^ cells compared to N1199^0^ cells (Fig. 5, SD + Rapamycin, *P*-value 0.0002). Similar to previous reports (Torggler *et al*. 2017), rapamycin treatment induced autophagy in the N1199^0^ cells without any associated vacuolar fragmentation, resulting in a more intense but punctate GFP signal (Fig. 5). Due to the low resolution of the above assays, we cannot sufficiently determine trafficking between different autophagosomal compartments. Nevertheless, our results are broadly consistent with the interaction between autophagy and the level of N1199. However, the directionality of the autophagy-N1199 level interaction—autophagy affects the level of N1199 or vice versa—remains to be determined.

In summary, in contrast to N1199, 20S (26 strains) and 23S (14 strains) are found less frequently in the 100-genomes strains (manuscript in preparation). There are 4 competing hypotheses for the near-universal frequency of N1199 vs the much lower frequencies of 20S and 23S. First, N1199^lo^ may have invaded *S. cerevisiae* before 20S and 23S. Second, the N1199^lo^ loss rate may be less than that of 20S and 23S. Third, while all 3 narnaviruses are cytoplasmically inherited, N1199^lo^ inheritance may be more efficient than that of 20S and 23S. Finally, while neither 20S, 23S nor N1199^lo^ are known to affect host fitness, potential deleterious effects on host fitness of N1199^lo^ may be less than that of 20S and 23S; conversely, potential beneficial effects on host fitness of N1199^lo^ may be greater than that of 20S and 23S.

Relative to 20S and 23S, N1199 has both similarities and differences. First, N1199^hi^ loss in YJM1199 is frequently accompanied by loss of 20S and/or 23S, which implies that host pathways required for maintenance of all 3 narnaviruses overlap, at least in part; autophagy is likely 1 such pathway. Second, 20S and 23S are present in low levels in YPD-grown cultures and the same is true for N1199 in 98 of the 100-genomes strains. However, in YPD-grown parental YJM1199, N1199 copy number is high while 20S and 23S copy numbers are low, which implies different mitotic growth copy number regulation of N1199 vs 20S and 23S, in at least 1 genetic background. Finally, while we did not examine N1199 copy number in sporulation media, 20S and 23S copy numbers increase in sporulation media. For example, a previous study showed that 20S, when present in strains, accumulated to high levels during sporulation; notably, the 20S^0^ strain Y55 did not accumulate a narnavirus-sized species during sporulation (Garvik and Haber 1978). In this context, the 20S^0^ strain Y55 is isogenic with YJM627 (N1199^lo^ 20S^0^ 23S^0^) (Supplementary Table 3), 1 of the 100-genomes strains (Strope, Skelly, *et al*. 2015). Thus, in contrast to 20S and 23S, in at least 1 genetic background, N1199 does not accumulate to high levels during sporulation. This result implies different sporulation copy number regulation of N1199 vs 20S and 23S, which may in part explain why N1199, despite its ubiquity, has not been previously described.

Due to sequence quality near the ends of N1199, we were not confident in our ability to construct an N1199 launching vector. In addition, due to the sporulation defect of *HO* YJM1199 N1199^hi^ and all *HO* YJM1199 segregants being N1199^0^, we were not able to introduce N1199^hi^ into N1199^0^ strains. Thus, we were not able to determine whether the level of N1199 in the parental YJM1199 N1199^hi^ was due to host genome sequences or N1199 genome sequences, or a combination of both. That said, the YJM1199 N1199^hi^ vs YJM1199 N1199^0^ phenotypes, such as the sporulation and carbon source utilization defects of YJM1199 N1199^hi^, clearly show that N1199^hi^ is deleterious to both N1199 itself and to the YJM1199 host. Finally, as exemplified by these YJM1199 N1199^hi^ results, RNA virus-dependent phenotypes, if not known or considered, have 2 implications. First, undetected RNA viruses may confound genotype association analyses. Second, undetected loss of RNA virus(es), which may occur during growth on different carbon sources (such as maltose) or drug treatments (such as rapamycin), may confound phenotypic analyses.

## Supplementary Material

jkac337_Supplementary_Data

## Data Availability

The N1199 sequence and plasmids described in this work have been deposited in NCBI (Accession number OP764685) and Addgene (http://www.addgene.org/John_McCusker/), respectively. Supplemental material available at G3 online.

## Notes

*Communicating editor: B. Andrews*
