# Supplementary material for: A novel narnavirus is widespread in Saccharomyces cerevisiae and impacts multiple host phenotypes
Source: G3 (Bethesda). 2022 Dec 23;13(2):jkac337. doi: 10.1093/g3journal/jkac337 (PMC9911063; doi:10.1093/g3journal/jkac337)
Supplement: jkac337_Supplementary_Data [file jkac337_supplementary_data.zip › Figure_S1_G3-2022-403775.pdf]

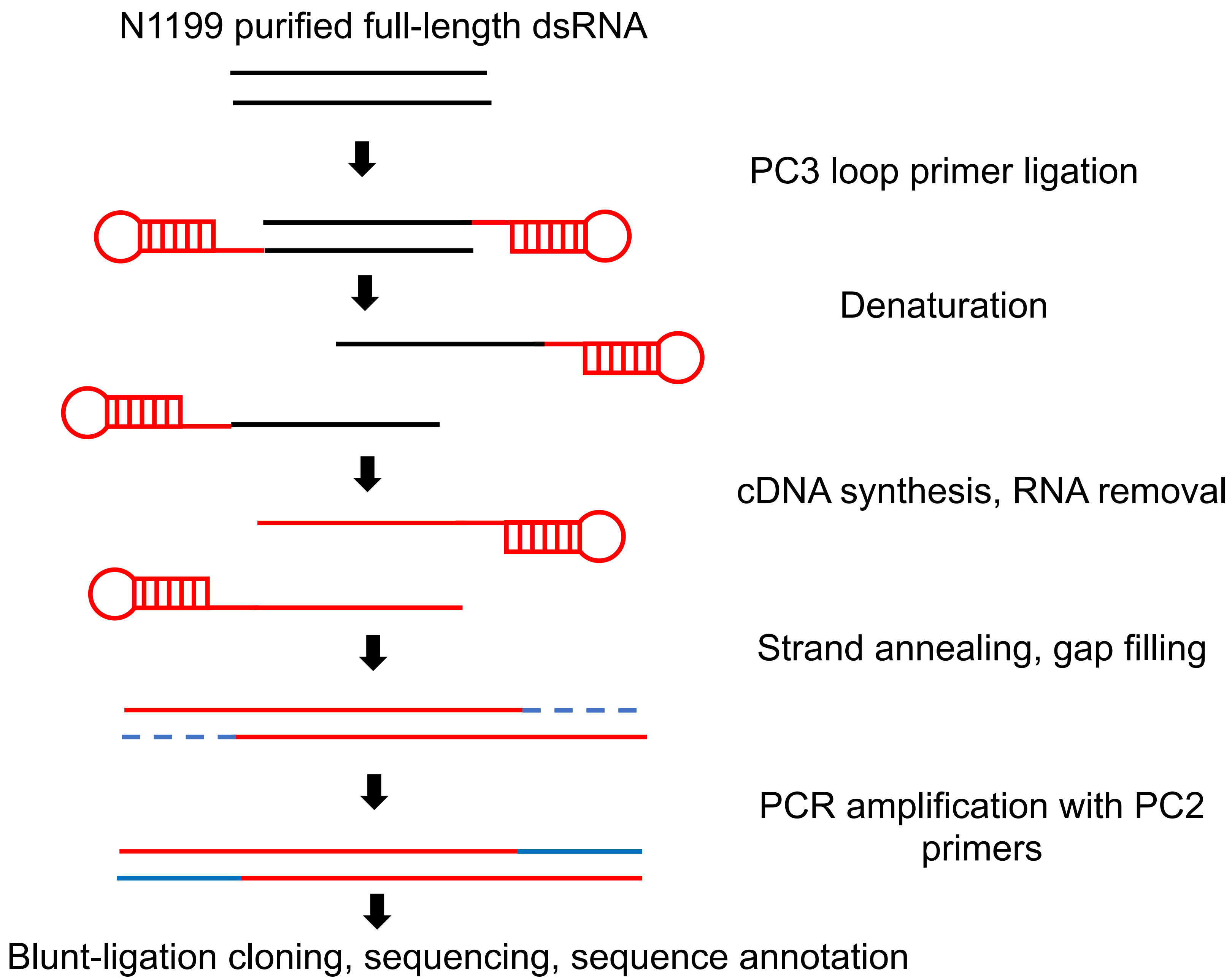
N1199 purified full-length dsRNA
PC3 loop primer ligation
Denaturation
cDNA synthesis, RNA removal
Strand annealing, gap filling
PCR amplification with PC2 primers
Blunt-ligation cloning, sequencing, sequence annotation
