# Supplementary material for: A novel narnavirus is widespread in Saccharomyces cerevisiae and impacts multiple host phenotypes
Source: G3 (Bethesda). 2022 Dec 23;13(2):jkac337. doi: 10.1093/g3journal/jkac337 (PMC9911063; doi:10.1093/g3journal/jkac337)
Supplement: jkac337_Supplementary_Data [file jkac337_supplementary_data.zip › Supplemental_Material_Legends_G3-2022-403775.docx]

**Supplemental Material Legends**

**Figure S1:** Schematic of N1199 sequence determination with FLAC. Refer to Methods for details. Red hairpins-PC3 loop primer, solid black line- RNA, solid red line-cDNA, blue lines (dashed)- gap-fill in after removal of RNA, blue lines (solid)- incorporation of PC2 sequence at the ends of the final full-length amplified N1199 sequence.

**Figure S2:** Phylogenetic analysis of N1199- and previously described narnavirus-encoded RNA-dependent RNA polymerase. The tree was created with pairwise alignments derived from PSI-BLAST (NCBI) of the N1199 ORF sequence, using a maximum likelihood method. Branch lengths vary according to the amino acid substitutions per site. Numbers in red on top of branches indicate the bootstrap confidence values for the clustered taxa. The *S. cerevisiae* N1199 is highlighted in blue. See Methods for additional details.

**Figure S3:** Cytoplasmic segregation of N1199 and other viruses. Segregation of N1199 and other viruses in meiotic progeny was analyzed by carrying out a cross between YSV698, a pAG36-containing haploid derivative of YJM682 (N1199^lo^), and YSV706, a pAG26-containing haploid derivative of YJM1463 (N1199^0^) to yield YSV709 (682 × 1463). RNA from the indicated strains was reverse transcribed and checked by PCR for presence/absence of various RNA virus species.

A-Parent strains (682- YSV698,1463-YSV706. Table S1) and the diploid (682 × 1463-YSV709, Table S1).

B- spore clones a-d (YSV 712-715, Table S1) from a representative tetrad derived from the sporulation of the diploid in panel A. PCRs—1-L-A, 2-L-BC, 3-20S, 4-23S,5-N1199. Primers for PCR analysis: L-A – L-A F2+R2, L-BC – L-BC F2+R2, 20S – RW307+RW308, 23S – 23S F2+R2, N1199– SV231+232, Mat locus–SV155+SV156+SV157 (Table S2).

**Figure S4**: Cell fractionation to determine sub-cellular localization of N1199. T=total, C=cytoplasmic, N=nuclei, Np=nucleoplasm. Refer to text and Supplementary information for details.

**Figure S5**: Analyzing the effect of amyloid prion curing and mitochondrial DNA loss on N1199.

A) Nucleic acid from the YJM1199 N1199^hi^ parent strain (ρ^+^) was compared with two independently derived ρ^0^ derivatives (YSV679,680, Table S1) on 1.3% TAE agarose gel. Loss of the mitochondrial genome has no effect on N1199^hi^. Lanes are from the same gel.

B) Gel analysis of RNA from YJM1199 cultures grown in YPD ± 5mM guanidine hydrochloride (Gdn-HCl) for 2 days at 30ºC to test the effects of curing amyloid prions, such as [PSI+], on N1199 levels.

C) Phenotypic test for [GAR+] in YJM1199. Cultures of YJM1199 (N1199^hi^) and four independent N1199-cured isolates (N1199^0^, Top to bottom YSV765-768, Table S1) were tested for growth on YP plates with 2% dextrose, 2% glycerol and 2% glycerol + 0.05% glucosamine.

**Figure S6:** Effect of sporulation and SKI1 overexpression on N1199 in YJM1199.

A: Representative agarose gel and RT-PCR analysis after Ski1 overexpression. SKI1 overexpression was induced in pSV41-transformed parental YJM1199 N1199^hi^, and total nucleic acid analysis was performed on 12 independent isolates.

Top- Gel analysis showing presence/absence of N1199^hi^ in 12 SKI1-overexpressing isolates. Note the absence of N1199^hi^ in lanes 1, 9, 10. Asterisk indicates a non-specific dye band.

Bottom- PCR analysis of isolates 1, 9, and 10 from top panel. YJM1199 N1199^hi^ (1199) was included as a positive control. PCRs a-20S,b-23S,c-N1199. Primers for PCR analysis: 20S – RW307+RW308, 23S – 23S F2+R2, N1199– SV231+232, Mat locus–SV155+SV156+SV157 (Table S2). MW- DNA molecular weight marker.

B: Representative gel and PCR analysis of YJM1199 N1199^hi^ and a single 4-spore tetrad obtained by sporulation of YJM1199 N1199^hi^. Gel (top) and (bottom) PCR analysis of N1199, 20S and 23S. Asterisk (*) denotes non-specific dye band.

**Figure S7**: Effect of galactose induction on N1199 abundance. YJM1199 transformed with empty vector (lanes 2-4) were induced with galactose as described in Methods. Subsequently samples were analyzed in 1.3% agarose TAE gel to check for the presence/absence of dsRNA species. Lane 1 is uninduced (no galactose treatment)

**Figure S8:** N1199^hi^ vs. N1199^0^/N1199^lo^ phenotypes in the YJM1199 background. A) Cultures of YJM1199 (N1199^hi^) and four independent N1199-cured isolates (N1199^0^, Top to bottom YSV765-768, Table S1) were tested. MGP-Methyl glucopyranose B) YJM1199 (N1199^hi^) compared against independent N1199^0^ and N1199^lo^ derivatives (Top to bottom: YJM1199, YSV765, YSV 790-793). Base of all media is YP.

**Figure S9:**  Reduced colony forming units of YJM1199 on media containing non-preferred carbon sources. A) YPD, Ara- YP + Arabinose, Mal- YP + Maltose

B) YP (no added carbon source, negative control) vs YPD

**Figure S10:** RT-PCRs to detect loss of N1199 upon A) PDE1 overexpression and B) Rapamycin treatments

**Table S1:** List of strains used in this study

**Table S2:** List of primers and plasmids used in this study

**Table S3:** PCRs to detect N1199 in the 100-genomes strains. PCRs were conducted with five independent primer pairs, using cDNA derived from total RNA, as described in Materials and Methods.

**Table S4:** Rate and frequency estimation of N1199 loss.
