# Supplementary figures and images for: A novel narnavirus is widespread in Saccharomyces cerevisiae and impacts multiple host phenotypes
Source: G3 (Bethesda). 2022 Dec 23;13(2):jkac337. doi: 10.1093/g3journal/jkac337 (PMC9911063; doi:10.1093/g3journal/jkac337)

**A**

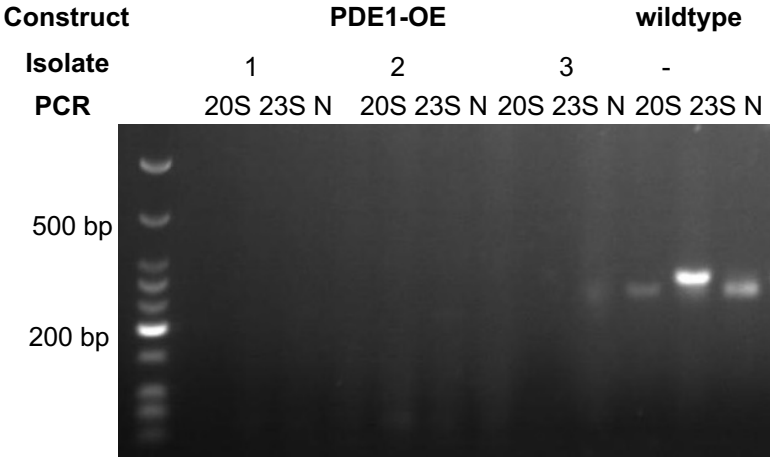

**B**

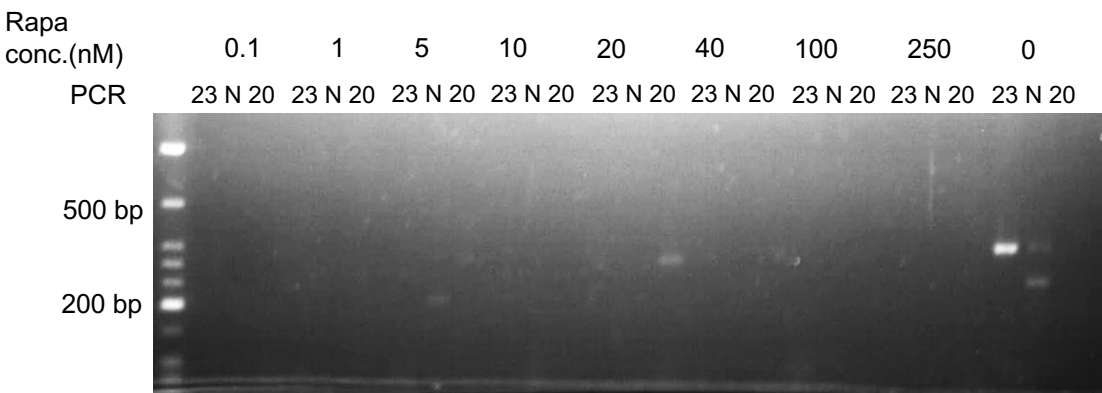

Supplement: jkac337_Supplementary_Data [file jkac337_supplementary_data.zip › Figure_S10_G3-2022-403775.pdf]

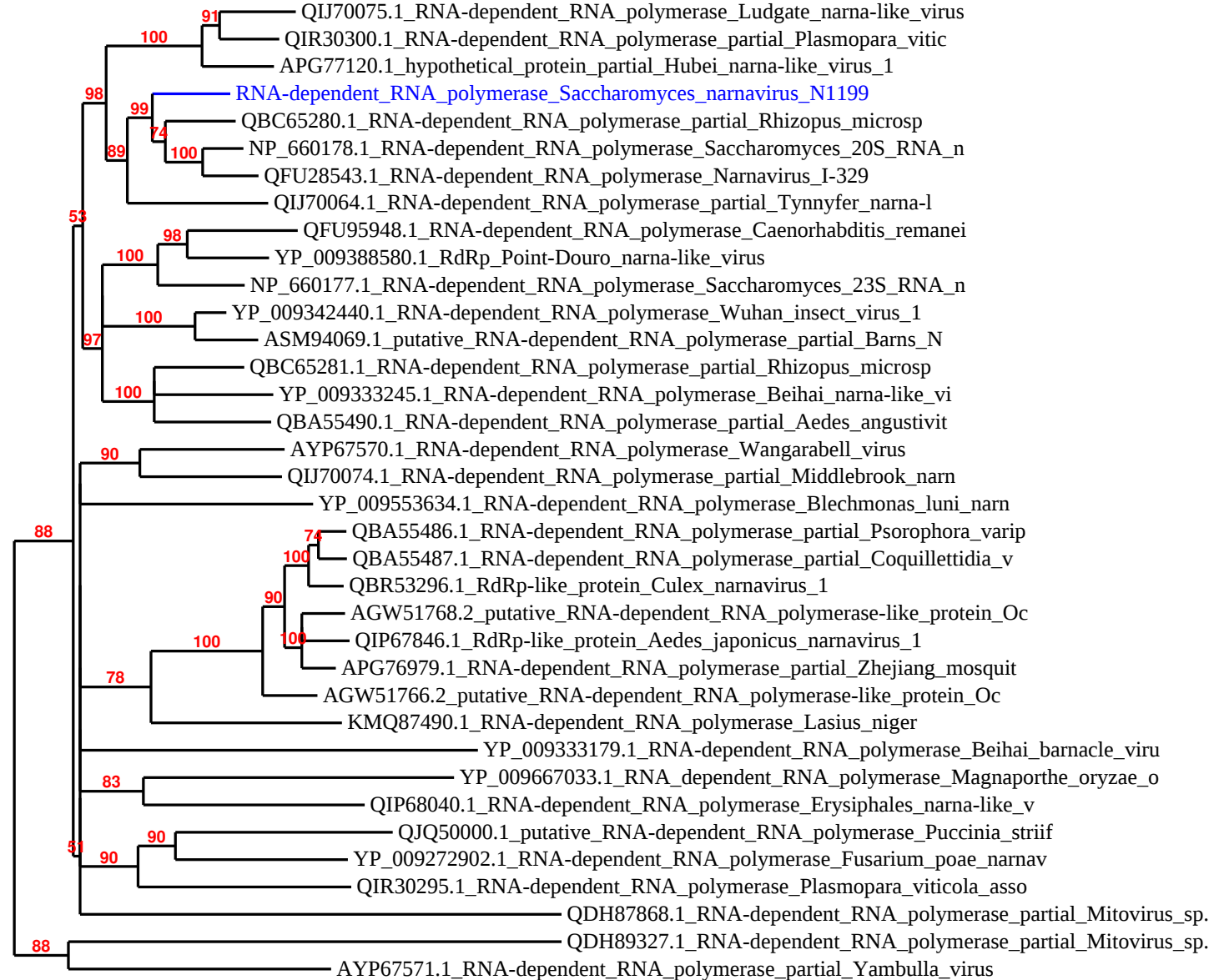

Supplement: jkac337_Supplementary_Data [file jkac337_supplementary_data.zip › Figure_S2_G3-2022-403775.pdf]

A.

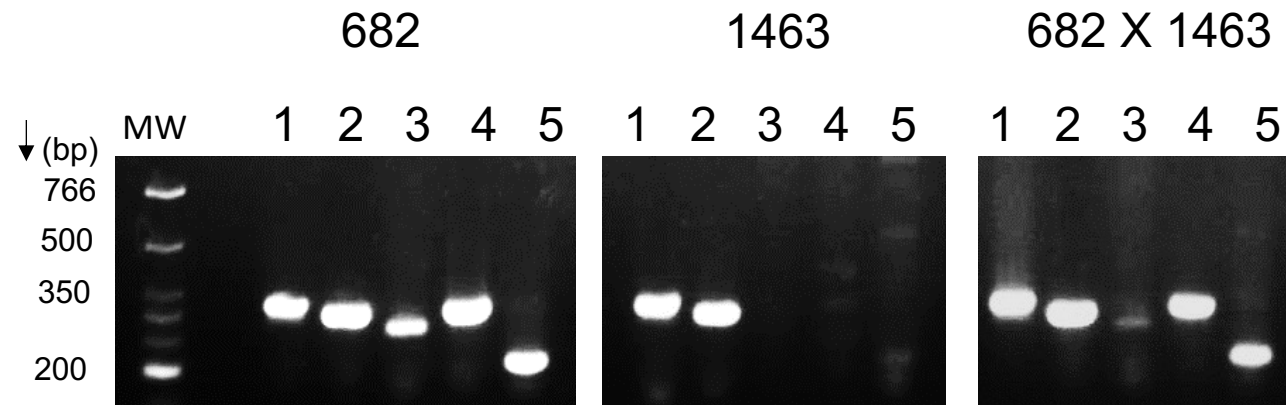

B.

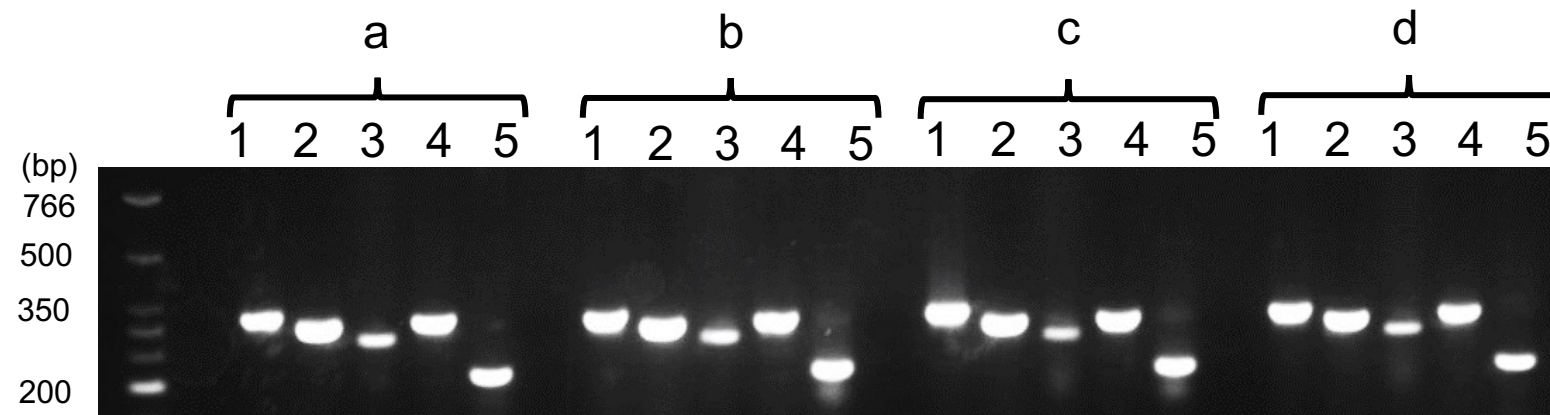

Supplement: jkac337_Supplementary_Data [file jkac337_supplementary_data.zip › Figure_S3_G3-2022-403775.pdf]

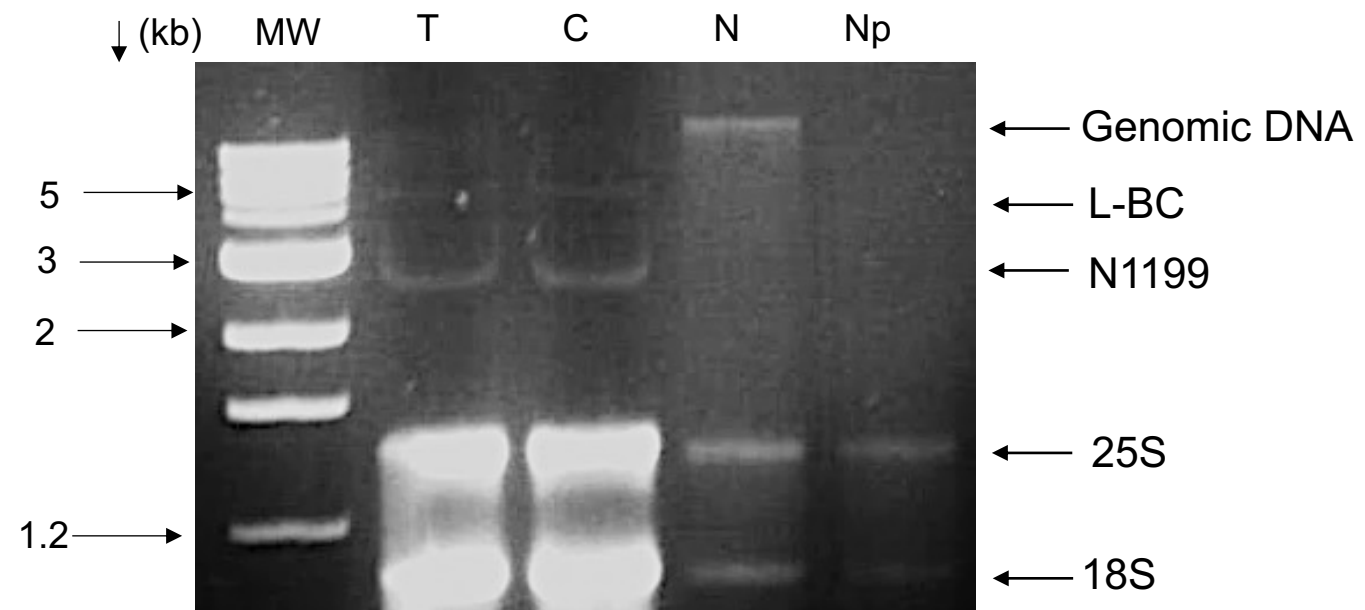

Supplement: jkac337_Supplementary_Data [file jkac337_supplementary_data.zip › Figure_S4_G3-2022-403775.pdf]

A.

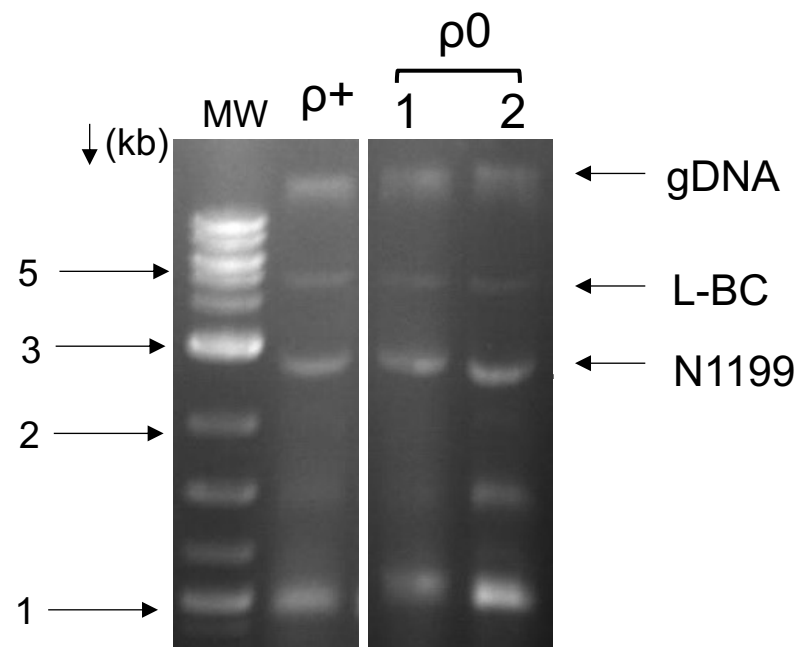

B.

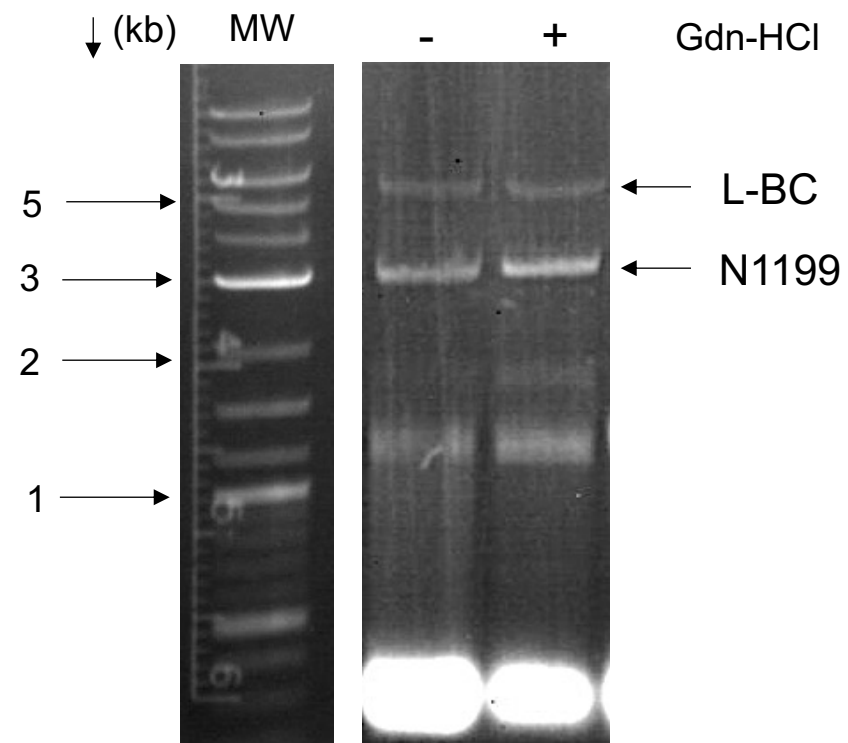

C.

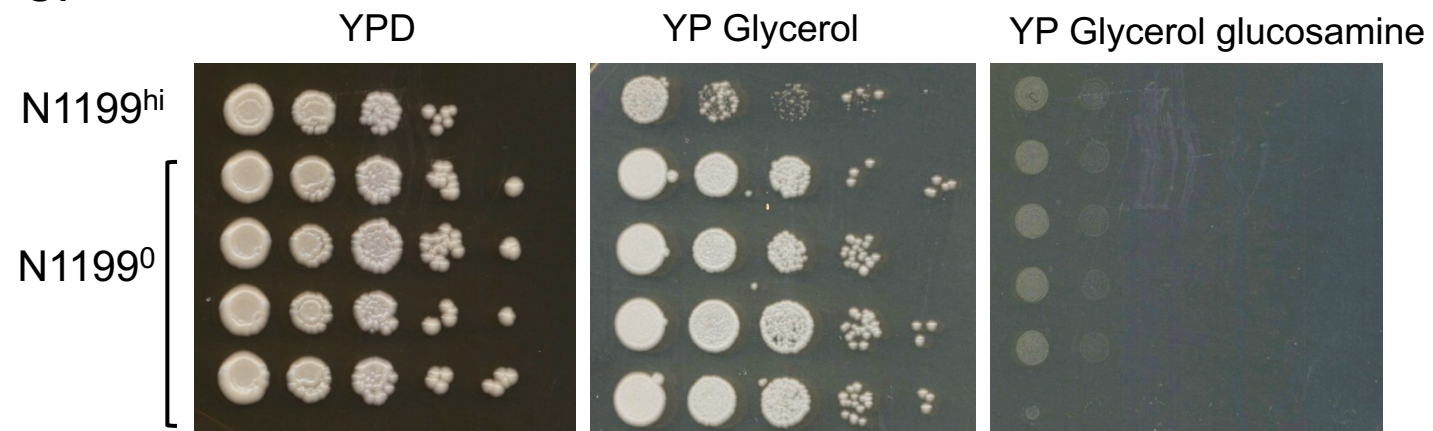

Supplement: jkac337_Supplementary_Data [file jkac337_supplementary_data.zip › Figure_S5_G3-2022-403775.pdf]

A.

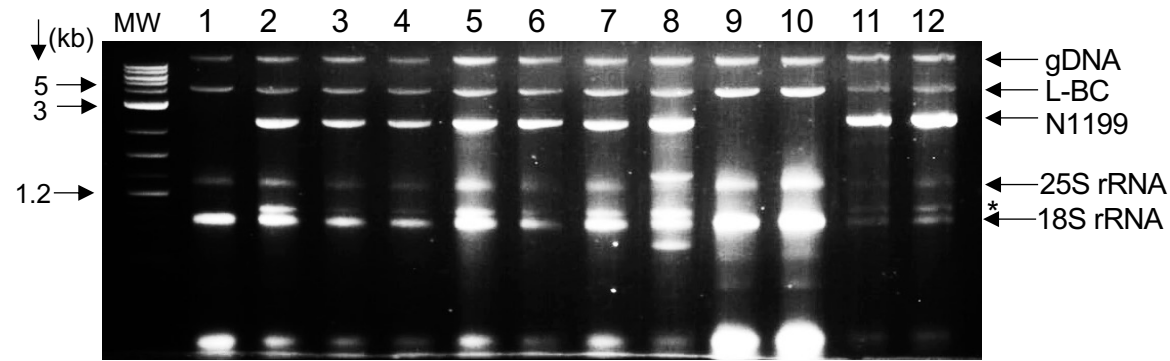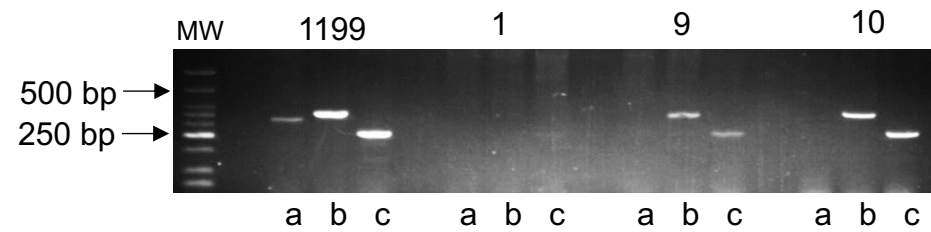

B.

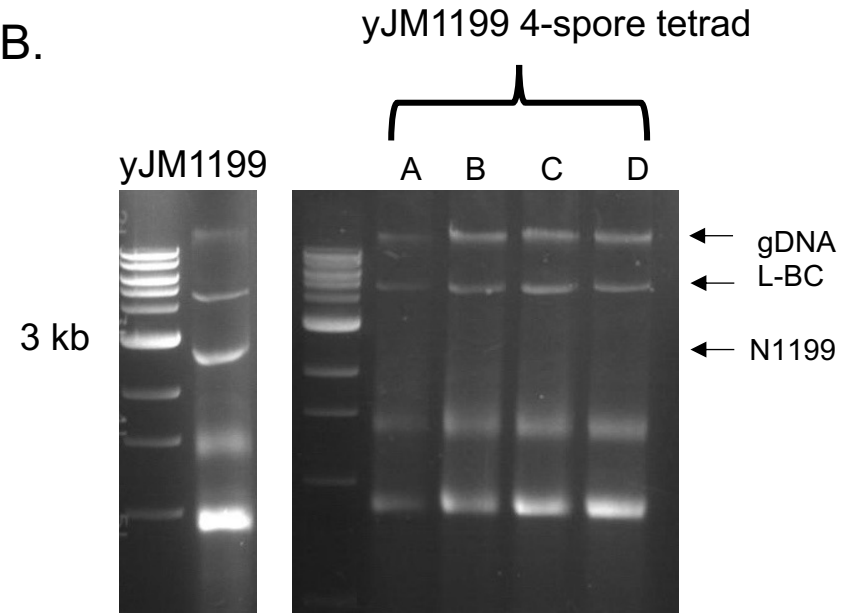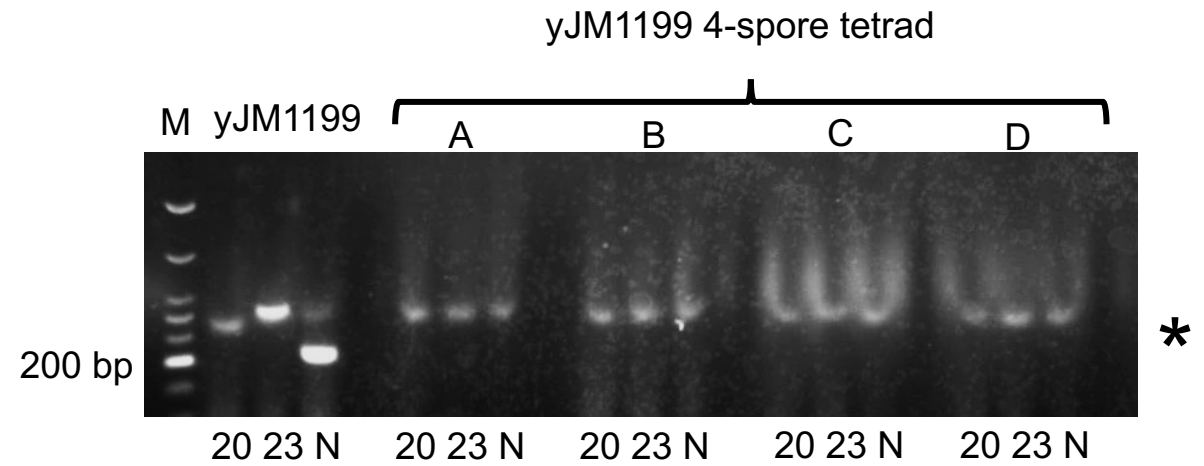

Supplement: jkac337_Supplementary_Data [file jkac337_supplementary_data.zip › Figure_S6_G3-2022-403775.pdf]

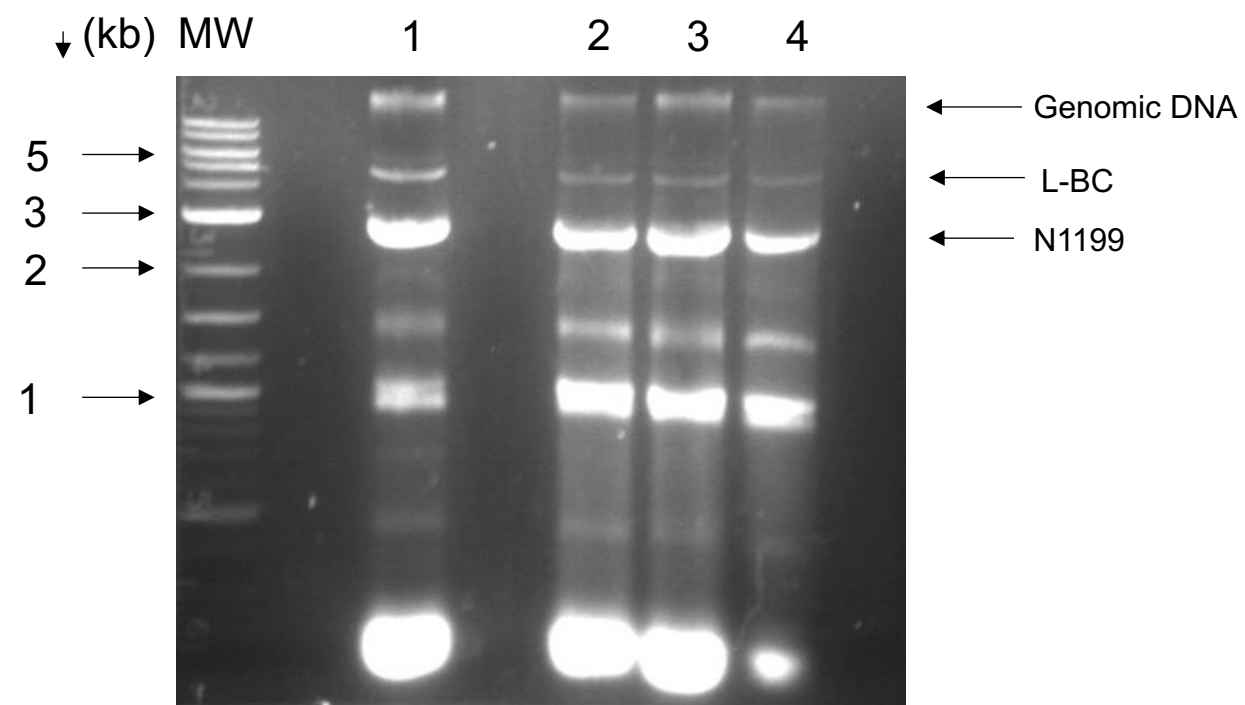

Supplement: jkac337_Supplementary_Data [file jkac337_supplementary_data.zip › Figure_S7_G3-2022-403775.pdf]

A

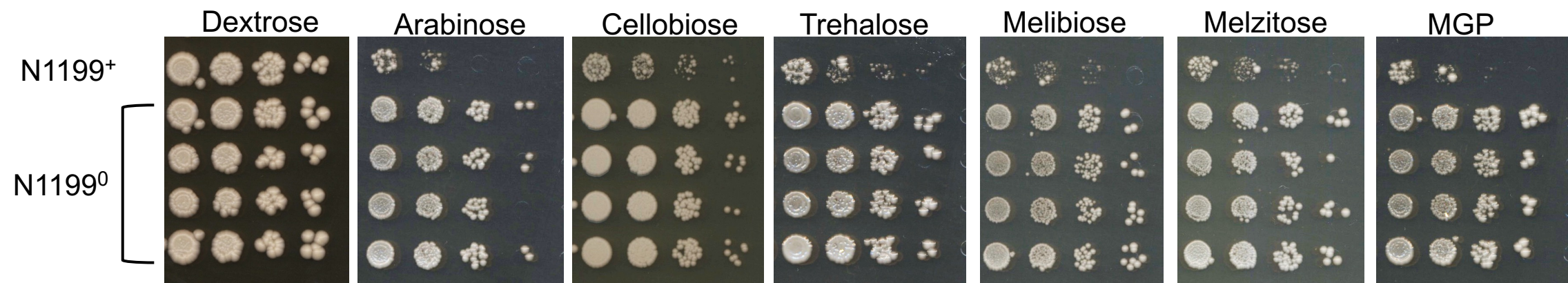

B

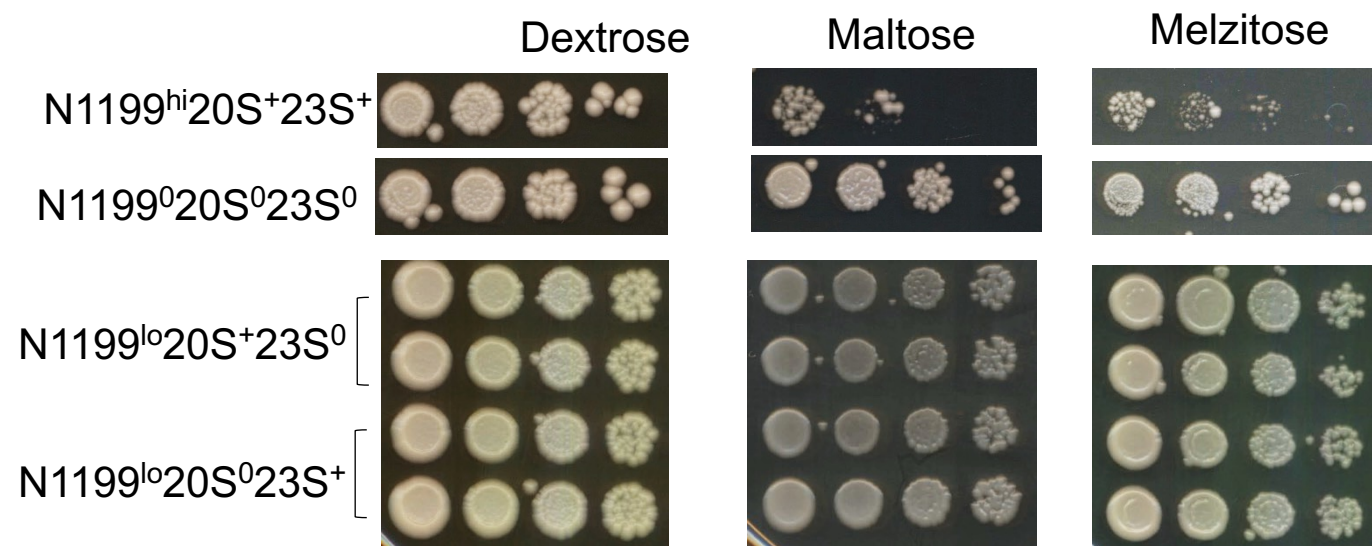

Supplement: jkac337_Supplementary_Data [file jkac337_supplementary_data.zip › Figure_S8_G3-2022-403775.pdf]

A

YPD

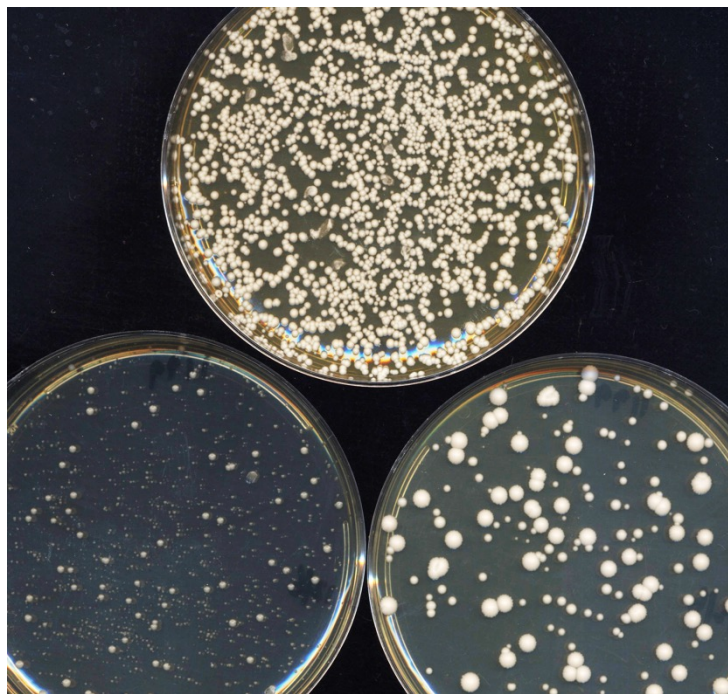

Ara

Mal

B

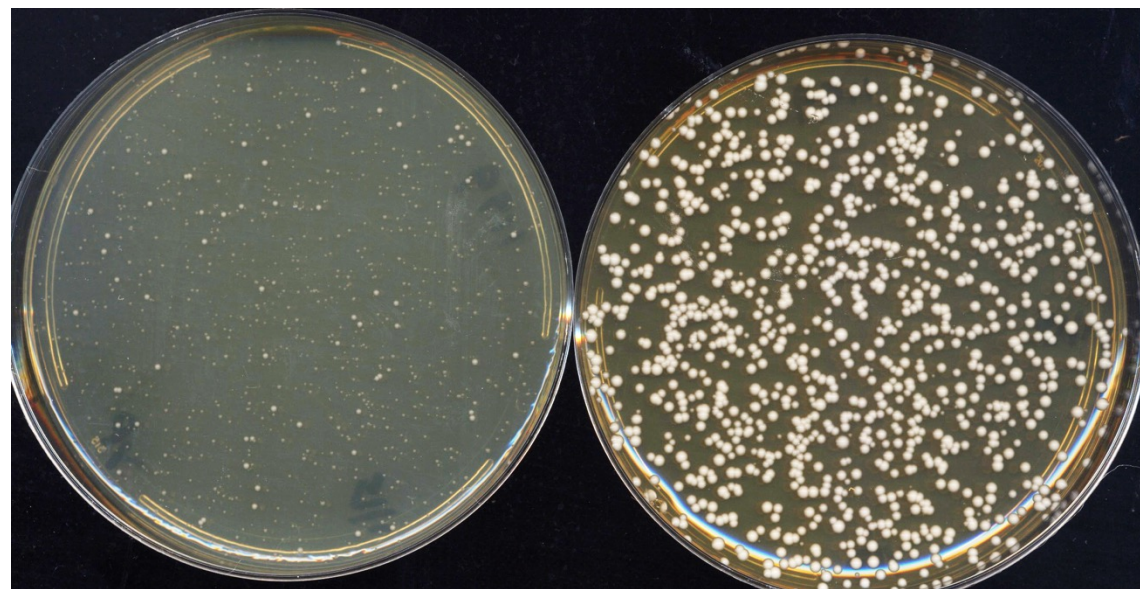

YP

YPD

Supplement: jkac337_Supplementary_Data [file jkac337_supplementary_data.zip › Figure_S9_G3-2022-403775.pdf]
